# Mitomycin-C-Induced TTP/HUS Treated Successfully with Rituximab: Case Report and Review of the Literature

**DOI:** 10.1155/2013/130978

**Published:** 2013-05-12

**Authors:** Gunjan Shah, Hanah Yamin, Hedy Smith

**Affiliations:** ^1^Tufts Medical Center, 800 Washington Street, P.O. Box 245, Boston, MA 02108, USA; ^2^Jordan Hospital, 275 Sandwich Street, Plymouth, MA 02360, USA

## Abstract

Microangiopathic hemolytic anemia (MAHA), thrombocytopenia, fever, renal failure, and neurologic symptoms comprise the cardinal features of thrombotic thrombocytopenic purpura and hemolytic uremic syndrome. Etiologies can include medications, infections, cancers, or transplantation. We present a patient with a history of rectal cancer treated with mitomycin-C who developed MAHA, acute kidney injury, and thrombocytopenia 6 months after completing therapy and to did not respond the plasmapheresis or steroids. She was treated with four weekly doses of rituximab with full recovery.

## 1. Introduction

Thrombotic thrombocytopenic purpura (TTP) and hemolytic uremic syndrome (HUS) encompass a spectrum of thrombotic microangiopathies classically associated with microangiopathic hemolytic anemia, thrombocytopenia, fever, renal failure, and neurologic symptoms. Further classification occurs based on the predominance of the cardinal features (with a greater degree of renal failure suggesting HUS and neurologic disease suggesting TTP), associated symptoms (diarrhea with typical HUS), level of the serum metalloprotease ADAMTS13 (low in TTP), and etiology. Secondary etiologies can include medications, infections, cancers, or transplantation. Several chemotherapeutic agents including mitomycin-C and gemcitabine and targeted therapies including bevacizumab and sunitinib have been implicated. We describe the case of a woman with mitomycin-C- (MMC-) induced TTP treated successfully with rituximab after failing a course of plasmapheresis and steroids. 

## 2. Case Presentation

A 73-year-old woman was referred for hematologic evaluation when she was found to be anemic and thrombocytopenic on preoperative labs for a hernia repair. She was found to have evidence of microangiopathic hemolytic anemia with acute kidney injury and admitted to her local hospital where she was transfused for a hemoglobin of 7.1 g/dL. She was transferred to our hospital for consideration of plasmapheresis. On admission, her hemoglobin was 11 g/dL, platelet count 74,000 /uL, creatinine 1.48 mg/dL, lactate dehydrogenase (LDH) 372 IU/L, and haptoglobin 2 mg/dL. D-dimer was mildly elevated and fibrinogen was normal making DIC unlikely. Her systolic blood pressure was elevated to 160, which had previously been normal. Past medical and surgical history included squamous cell anal carcinoma diagnosed 10 months previously and being treated with a diverting loop colostomy, radiation therapy, 5-FU, and mitomycin-C. She also had macular degeneration, paroxysmal atrial fibrillation, hypothyroidism, osteopenia, and back pain attributed to radiation neuropathy. Family history was significant for Parkinson's disease in her father and breast cancer in an aunt, but no hematologic diseases. She denied any tobacco, alcohol, or drug use and had previously worked in child care without any exposure history. Medications on admission included levothyroxine, metoprolol, warfarin, calcitonin, fish oil, vitamin D, calcium, and a multivitamin.

She was thought to have TTP and started on daily plasma exchange for three days with no improvement in her platelet count and minimal evidence for use with chemotherapy-induced TTP; so it was discontinued. Prednisone 80 mg daily was then started along with continued red blood cell and platelet transfusions. A disintegrin and metalloproteinase with a thrombospondin type 1 motif, member 13 (ADAMTS-13) level was checked; the activity level was 77% and no inhibitor was detected. With the steroids, her platelet count remained at 30,000, and her prednisone was tapered to 70 mg daily. At the time of first outpatient followup, she complained of edema in her face and legs, as well as proximal thigh muscle weakness and fatigue such that she was unable to ambulate or climb stairs. She had one nose bleed after discharge for which she was transfused several units of blood. As the steroids were not elevating her platelet count and she had significant side effects, these were tapered. A bone marrow biopsy was performed to rule out other causes of thrombocytopenia, which revealed hypocellularity related to her prior pelvic radiation exposure. At this point one month after presentation, her hemoglobin was 14 g/dL (maintained with several transfusions), platelet count 18,000 /uL, creatinine 2.14 mg/dL, haptoglobin 2 mg/dL, and LDH 614 IU/L.

At that point, the etiology of her microangiopathy was felt to be drug induced. She had received her last dose of mitomycin-C six months earlier with a total dose of 32.5 mg. In addition, she had had one intraocular administration of bevacizumab three months prior to presentation for macular degeneration. Given the known association with MMC, this was thought to be the offending drug. 

**Figure 1:**
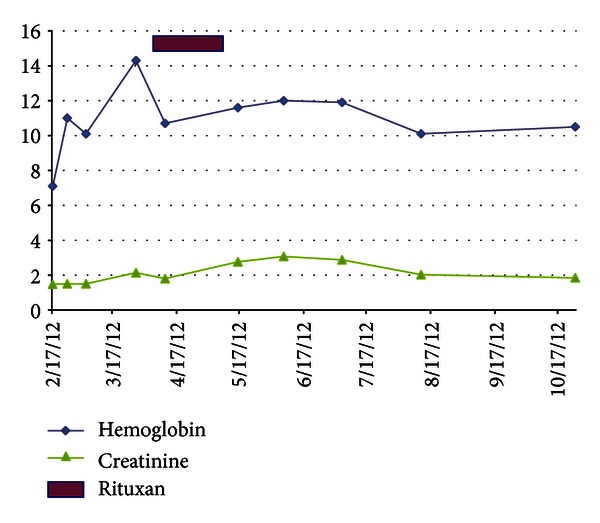
Hemoglobin and creatine response with rituximab intervention.

**Figure 2:**
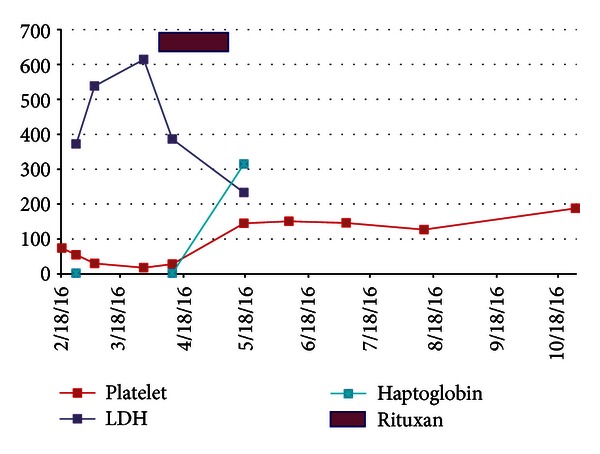
Platelet count, LDH, and haptoglobin response with rituximab intervention.

She was deemed unresponsive to plasmapheresis and steroids and, therefore, required an alternative treatment. We treated her with weekly doses of rituximab 375 mg/m^2^ for 4 doses, which she tolerated well. Two weeks after completion, her hemoglobin was 11.6 g/dL, platelet count 145,000 /uL, creatinine 2.76 mg/dL, haptoglobin 315 mg/dL, and LDH 233 IU/L. Her peripheral smear showed no evidence of hemolysis and resolution of microangiopathic changes. She remains in remission from the microangiopathic hemolytic anemia and thrombocytopenia now 11 months after her initial presentation, though her renal function has still not returned to her baseline (Figures 1 and 2, Table 1). 

## 3. Discussion

Mitomycin-C is used to treat many types of solid tumors, including gastric, pancreatic, bladder, and breast cancers, with a new resurgence of use in colorectal tumors. Side effects can include myelosuppression, nausea, vomiting, and fever, with TTP/HUS occurring in less than 15% of the patients [1]. 

Our patient's case had some unusual features. Her symptoms began 10 months after completion of her therapy with a cumulative dose of 32 mg/m^2^ of MMC. While reports of MMC TTP/HUS state that the onset is usually between 4 and 9 weeks of completion of chemotherapy, symptoms may occur up to 15 months later [2]. In addition, the incidence tends to be related to total dose, with most patients having a cumulative dose of greater than 40 mg/m^2^ [3]. Treatments for mitomycin-C induced TTP are largely supportive. Plasmapheresis has been beneficial in only a few cases [4], but steroids have been reported to be helpful in about 30% of patients [5]. In our patient's case, the microangiopathic hemolytic anemia and thrombocytopenia did not respond to plasmapheresis or high-dose steroids, and instead, she developed a steroid myopathy with significant quality of life decrement. Hong et al. [6] published a case report of the successful use of rituximab, which we based our treatment on. We could find only one other case report of rituximab given for MMC-induced TTP/HUS, but were not able to access the article [7].

The mechanism of action of rituximab in this situation is unclear. Toxic-nonspecific insults to the microvasculature are possibly the etiology of chemotherapy-induced TMA [8] with direct endothelial cell injury reproduced in an animal model of mitomycin-induced HUS [9]. One postulated mechanism is the suppression of the generation of the ADAMTS13 inhibitor, while another theory is that the abnormal immune response to von Willebrand factor-cleaving protease is important for the development of acquired TTP [10]. However, our patient did not have an inhibitor so it is more likely that altering her immune function played a role in her response. 

It remains unclear whether the degree of symptoms at presentation dictates the likelihood of response to rituximab. In our case, four weekly treatments at the standard dose of 375 mg/m^2^ were enough to produce a response. The duration of response or risk of relapse is unclear as well, but the possibility of remission and ease of administration makes rituximab a good option for refractory patients. 

## 4. Conclusion

As mitomycin-C is increasingly used to treat rectal cancers, a noninsignificant portion of patients may present with TTP/HUS related to their chemotherapy. Steroids remain a first-line option, but the use of rituximab can be considered in those not responding to initial therapy. 

## Figures and Tables

**Table 1:** Measured lab values in patient's course.

Date	Hemoglobin	LHD	Haptoglobin	Platelet	Creatinine	Intervention
2/17/12	7.1			74	1.48	
2/24/12	11	372	2	55	1.5	
2/26/12	7.9	396		37	1.59	Prednisone 80 mg started
2/29/12	9.2	440		47	1.68	Plasmapheresis daily ×3
3/4/12	10.1	538		30	1.5	Prednisone 60 mg × 3 wk
3/28/12	14.3	614		18	2.14	Prednisone taper
4/11/12	10.7	386	2	28	1.8	Rituxan weekly ×4 started
5/16/12	11.6	233	315	145	2.76	
6/7/12	12			151	3.07	
7/5/12	11.9			146	2.88	
8/12/12	10.1			127	2.03	
10/25/12	10.5			188	1.85	
